# User Experiences of Electronic Controlled Substance Surveillance Among Health Care Professionals in a Large Academic Hospital: Cross-Sectional Survey

**DOI:** 10.2196/94815

**Published:** 2026-08-26

**Authors:** Annika Häkkinen, Hanna M Tolonen, Tinja Lääveri, Johanna Viitanen, Kaisa Savolainen, Mia Sivén

**Affiliations:** 1 Faculty of Pharmacy, University of Helsinki, Viikinkaari 5 E, Helsinki, Uusimaa, Finland, +358 29 41 911; 2 HUS Pharmacy, Helsinki University Hospital, Helsinki, Uusimaa, Finland; 3 Department of Computer Science, Aalto University, Espoo, Uusimaa, Finland; 4 Infectious Diseases, Inflammation Center, Helsinki University Hospital and University of Helsinki, Helsinki, Uusimaa, Finland; 5 Helsinki Institute of Sustainability Science, HELSUS, Helsinki, Uusimaa, Finland

**Keywords:** controlled substance, electronic health record, electronic narcotic consumption card, health care professional, usability, user experience

## Abstract

**Background:**

Controlled substances are commonly used as analgesics or to treat neurodevelopmental disorders in health care, but they also pose a risk of drug abuse and diversion. Therefore, the distribution and handling of controlled substances are strictly regulated, and hospital pharmacies are required to manage their surveillance in hospital settings. Between 2018 and 2021, a large Finnish academic hospital implemented a new electronic health record system enabling the digitalization of paper-based controlled substance surveillance. By November 2023, the electronic narcotic consumption card (eNCC) had been implemented in 84 wards. Although hundreds of health care professionals (HCPs) use the eNCC daily, our understanding of the user experience (UX) with this process is limited.

**Objective:**

This study aimed to characterize UX with the eNCC and compare it across different dosage forms. Additionally, we aimed to assess whether the end user’s professional role, years in practice, and years of electronic health record system use affect UX and how investigations and error corrections in controlled substance surveillance have evolved since the implementation of the eNCC.

**Methods:**

An electronic survey was conducted among HCPs working in wards and the hospital pharmacy in May 2024. The survey included background questions, statements using a 5-point Likert scale, and 1 open-ended question. This study was reported in accordance with the CROSS (Consensus-Based Checklist for Reporting of Survey Studies) guidelines.

**Results:**

There were 599 respondents (n=522, 87.1% nurses; n=21, 3.5% physicians; n=56, 9.3% pharmacists) to the survey during the 3-week response period. Our main finding was that HCPs preferred the eNCC process; 80.9% (484/598) of respondents disagreed when asked whether they were willing to return to the paper-based process. Additionally, HCPs considered the eNCC to be effortless in daily work for both solid (491/578, 84.9%) and liquid (418/578, 72.3%) dosage forms. However, the eNCC process for solid dosage forms was reported to be more straightforward and faster than that for liquid ones. Both nurses and pharmacists reported conducting more investigations and error corrections immediately after eNCC implementation than during the most recent month. Nevertheless, ward pharmacists performed more investigations and corrections than registered nurses at both specified time points.

**Conclusions:**

Most HCPs had rather positive attitudes toward the eNCC, and they preferred it over a paper-based process. This research highlights the importance of understanding the varying levels of interaction with the eNCC among different professional roles.

## Introduction

Controlled substances such as opioids used as analgesics or in opioid agonist therapy or stimulants for attention-deficit/hyperactivity disorder are essential in patient care but carry risks of abuse and diversion, potentially leading to addiction, infections, or even death [1-6]. Diversion, the illegal acquisition of a patient’s medication by health care professionals (HCPs) for personal use, underscores the need for strict controlled substance regulation [7]. Health care organizations endeavor to prevent diversion through various measures, including technological solutions; surveillance systems; and individual-level practices in storage, prescribing, and administration [8]. Within public health care hospitals, hospital pharmacies are responsible for overseeing controlled substance surveillance in Finland [9,10].

Finland is internationally recognized as a leader in health care digitalization, with full electronic health record (EHR) coverage in specialized care since the early 2010s and nationwide e-prescribing established from 2010 to 2014 [11]. Despite this, controlled substance surveillance largely remained paper based, requiring manual entry of all relevant data into surveillance documents [12]. The implementation of the Epic-based Apotti EHR system between 2018 and 2021 enabled replacing the paper-based controlled substance surveillance process with an electronic one [12,13]. The electronic narcotic consumption card (eNCC) was developed to automatically extract controlled substance–related data from the EHR [12]. By November 2023, the eNCC had been implemented in 84 wards.

Digital technologies have the potential to reduce medication errors, save HCPs time, and strengthen controlled substance surveillance [6,12], but technology alone cannot eliminate diversion as process loopholes and work-around–prone steps persist [6]. In addition to being associated with end user dissatisfaction, ineffective workflows, and work-related stress and burnout [14-18], usability problems may lead to work-arounds [19,20] that in turn are associated with patient safety incidents [21-25]. Examining end users’ experiences is essential for identifying not only usability issues but also perceived and expected benefits [26,27], as well as technology acceptance [28]. Field data from the eNCC further indicate that workflows are becoming faster and traceability is improving, especially for tablets and capsules, while also revealing preexisting work-arounds that can be mitigated through training and design refinements [12].

Despite initial evidence suggesting that the eNCC improves documentation accuracy and workflow efficiency [12], little is known about the usability of the eNCC in routine clinical work or how HCPs’ experiences vary across medication types and user groups. To address this research gap, this study investigated the usability and user experience (UX) of the EHR-integrated eNCC and its implications for workflows and controlled substance oversight. Specifically, we aimed to (1) characterize overall UX with the eNCC; (2) compare UX across dosage forms; (3) assess how professional role, years in practice, and years of EHR system use relate to UX; and (4) determine whether data quality investigations and error corrections changed after eNCC implementation.

## Methods

### Study Setting and Design

Different HCPs have distinct responsibilities in the narcotic consumption card (NCC) process [12]. Physicians verify NCCs with their signatures before pharmacy approval and archiving, whereas nurses and pharmacists perform broader tasks, including medication administration and error investigation [12].

Experiences with the eNCC process were examined using a cross-sectional electronic survey, a common method in health care research to complement qualitative findings [29]. We reported our survey following the CROSS (Consensus-Based Checklist for Reporting of Survey Studies) guidelines [30].

### Survey Instrument

A survey questionnaire (Multimedia Appendix 1) comprising 4 themes was developed by a multidisciplinary group of experts in pharmacy, medicine, and UX research, as well as those with experience in the EHR system. Instead of using standardized usability questionnaires (eg, System Usability Scale and Usability Metric for User Experience), we developed a study-specific questionnaire. While the System Usability Scale was used in an earlier study by our team [12], it did not differentiate between the workflows examined in the present study.

Separate versions were created for physicians and for nurses and pharmacists to reflect their distinct roles in the eNCC process [12]. The core themes addressed (1) usability, (2) context-dependent use, and (3) attitudes and perceptions. These themes were based on established usability dimensions [31]. In addition, the questionnaire included demographic items such as professional background, department, hospital, years of professional experience, current working unit, and experience with the EHR system. The questionnaire was implemented using the REDCap software (Vanderbilt University) [32,33].

Questionnaire items were designed to reflect the 4 predefined themes and ensure comprehensive coverage of usability aspects. All professional groups were presented with 10 general UX statements rated on a 5-point Likert scale (“strongly disagree”-“strongly agree”), 1 net promoter score (NPS) item (0‐10) [34], and 1 open-ended question. The open-ended item captured qualitative feedback on the eNCC process and its development. Additional role-specific modules were included for nurses and pharmacists. These comprised statements related to dosage forms (ie, solids, such as tablets, capsules, and transdermal patches, and liquids, such as injections, infusions, and liquid oral preparations), reflecting common hospital formulary. To assess perceived workload effects among nurses and pharmacists, additional items were included to estimate the time spent on investigations and error corrections after eNCC implementation and during the most recent month.

Content validity was ensured through iterative refinement with a research group experienced in surveys and eNCC workflows. Face validity was supported by piloting (n=8) the questionnaire with representatives from each HCP group using a discussion-based approach consistent with cognitive interviewing techniques [35]; feedback from these sessions informed minor revisions. Pilot responses were excluded from analysis as the pilot aimed to evaluate question clarity and questionnaire usability rather than generate study data.

### Study Population, Recruitment, and Survey Distribution

The targeted population comprised physicians, registered nurses, practical nurses, and ward pharmacists across 84 hospital wards, as well as pharmacists at the HUS Helsinki University Hospital pharmacy. With ward staffing levels typically ranging from 30 to 70 HCPs, the estimated study population was 2520 to 5880 individuals. At the time of the survey, the eNCC was used across 14 specialized health care departments, all represented in the sample, and had been implemented for at least 6 months in every department.

This study was conducted at HUS Helsinki University Hospital, Finland, in May 2024. Survey invitations were distributed via head nurses and pharmacy supervisors, who received the link via email and forwarded it to all eligible HCPs in their units. The survey remained open for 3 weeks, during which 2 reminders were sent through the same intermediaries. This personalized approach to a defined population has been shown to improve online survey response rates [36].

### Data Preparation and Analysis

Before analysis, participants who selected “other” as their professional group were combined with registered nurses. Work experience categories were reduced from 5 to 3.

Data were analyzed using both qualitative and quantitative methods. For statistical analysis, nonparametric tests were applied. The Kruskal-Wallis one-way ANOVA was used to assess UX differences across professional groups, work experience, and years of EHR system use, with pairwise comparisons performed when significant differences were found. The Wilcoxon matched-pair signed-rank test compared the eNCC process for different dosage forms (solid vs liquid). The NPS was calculated as the percentage of promoters (ratings of 9‐10) minus the percentage of detractors (ratings of 0‐6) [34]. All statistical analyses were performed using the original 5-point Likert scale. For graphical presentation, Likert-scale responses were aggregated into 3 categories to improve the readability and interpretability of the figures. Analyses were conducted using Microsoft Excel and SPSS Statistics (version 28.0; IBM Corp), whereas open-ended responses were analyzed thematically in Microsoft Excel.

Item-level missing data were possible because individual responses were not mandatory, but REDCap prevented partially completed questionnaire submissions because respondents had to reach and confirm the final page before submitting the questionnaire. Missing data were not imputed and were handled using pairwise deletion such that each analysis included all available observations for the variables involved. Nonresponse error could not be evaluated as the target population was not fully identifiable and the response rate could not be determined precisely.

### Ethical Considerations

This study was conducted in accordance with Finnish regulations and the principles of the Declaration of Helsinki. According to the Finnish National Board on Research Integrity guidelines [37], no separate ethical review was required because participants were surveyed in their professional roles and no sensitive information was collected. Participation was voluntary, and electronic informed consent was obtained before respondents accessed the survey. All responses were anonymous and accessible only to the research team. HUS Helsinki University Hospital granted research approval (47/2024).

## Results

A total of 599 HCPs completed the survey, yielding a response rate of 10% (n=5880) to 24% (n=2520). The largest respondent group was registered nurses (n=511, 85.3%), followed by ward pharmacists (n=33, 5.5%), other pharmacists (n=23, 3.8%), physicians (n=21, 3.5%), and practical nurses (n=11, 1.8%; Table 1). Of the respondents, 30.1% (n=180) had 5 years of experience or less, whereas 41.7% (n=250) had over 16 years of work experience. Pharmacists and practical nurses generally reported less experience than physicians and registered nurses. Most participants (n=426, 71.1%) reported more than 3 years of system use.

**Figure 1.**
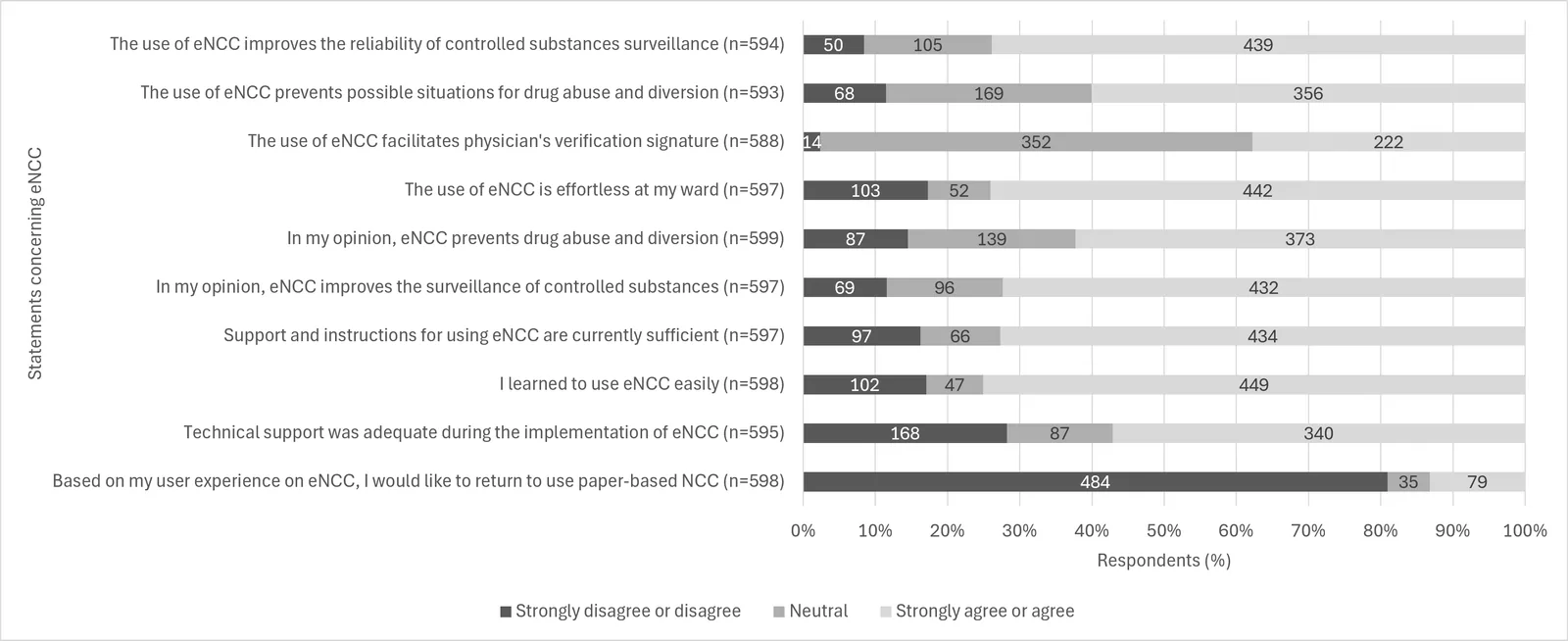
User experience statements concerning the electronic narcotic consumption card (eNCC) for health care professionals.

All respondents rated 10 usability statements on their experiences of the eNCC (Figure 1). Overall, perceptions were positive. Most respondents (484/598, 80.9%) disagreed with wanting to return to the paper-based NCC, whereas 13.2% (79/598) agreed. Most believed that the eNCC improved the reliability of controlled substance surveillance (439/594, 73.9%) and helped prevent drug abuse and diversion (373/599, 62.9%). Technical support was considered adequate both during implementation (340/595, 57.1%) and at the time of the survey (434/595, 72.9%). Most respondents reported that learning to use the eNCC was easy (449/598, 75.1%) and that its use was effortless on their wards (442/597, 74.0%). Views on whether the eNCC facilitated physician signatures were mixed: 59.9% (352/588) were neutral, and 37.8% (222/588) agreed.

**Table 1.** Background information on study participants (N=599).

	Physicians (n=21), n (%)	Registered nurses (n=511), n (%)	Practical nurses (n=11), n (%)	Ward pharmacists (n=33), n (%)	Other pharmacists (n=23), n (%)	Total, n (%)
Work experience (years)						
0‐5	6 (28.6)	142 (27.8)	7 (63.6)	14 (42.4)	11 (47.8)	180 (30.1)
6‐15	4 (19)	145 (28.4)	4 (36.4)	11 (33.3)	5 (21.7)	169 (28.2)
≥16	11 (52.4)	224 (43.8)	0 (0)	8 (24.2)	7 (30.4)	250 (41.7)
EHR^a^ system use (years)						
<1	2 (9.5)	21 (4.1)	2 (18.2)	3 (9.1)	8 (34.8)	36 (6.0)
1‐3	2 (9.5)	108 (21.1)	5 (45.5)	12 (36.4)	10 (43.5)	137 (22.9)
>3	17 (81)	382 (74.8)	4 (36.4)	18 (54.5)	5 (21.7)	426 (71.1)

a EHR: electronic health record.

**Figure 2.**
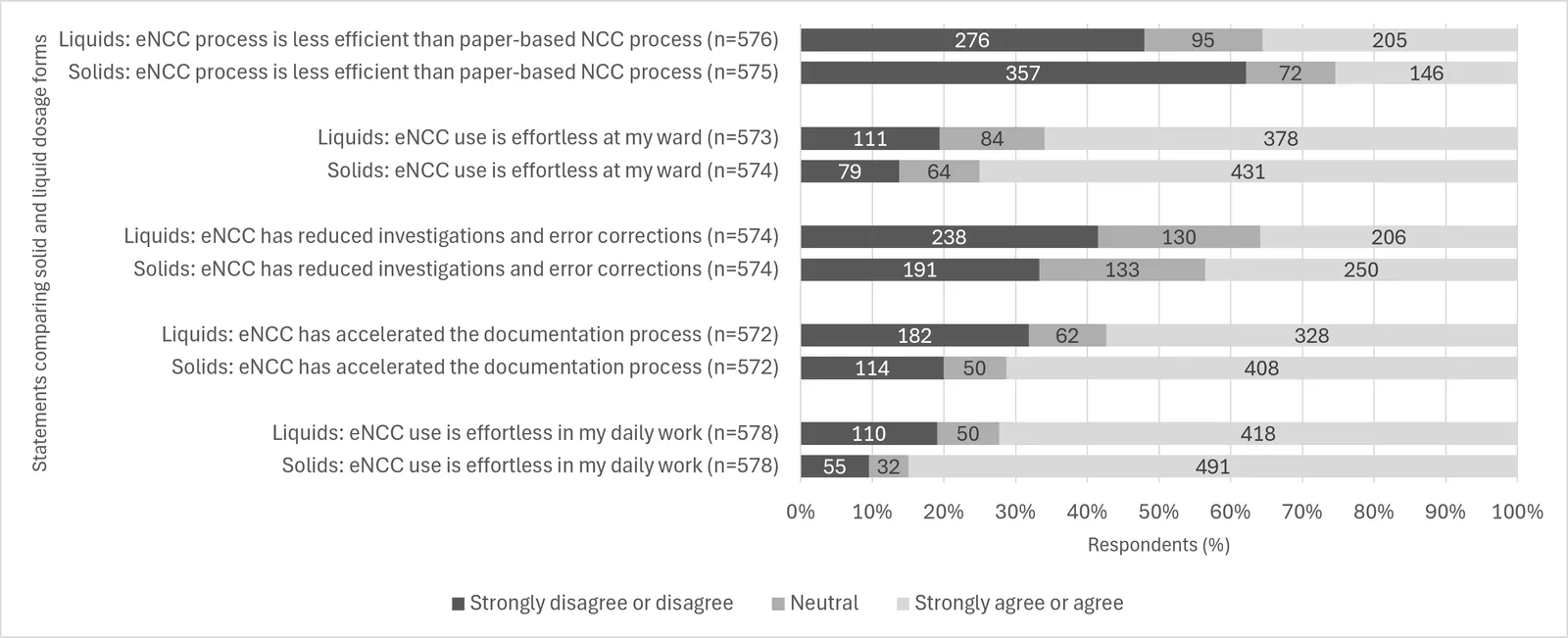
User experience with the electronic narcotic consumption card (eNCC) for tablets, capsules, and transdermal patches (solids) compared to injections, liquid oral preparations, and infusions (liquids) as reported by nurses and pharmacists.

Nonphysician respondents (578/599, 96.5%) were asked to rate 5 statements comparing eNCC processes for solid and liquid dosage forms (Figure 2). Solids included tablets, capsules, and transdermal patches, whereas liquids comprised liquid oral preparations, injections, and infusions. The process for solids was perceived as more straightforward, faster, and requiring fewer error corrections than that for liquids (*P*<.001). Satisfaction was high for both: 72.3% (418/578) agreed that using the eNCC for liquids was effortless compared with 84.9% (491/578) for solids.

**Figure 3.**
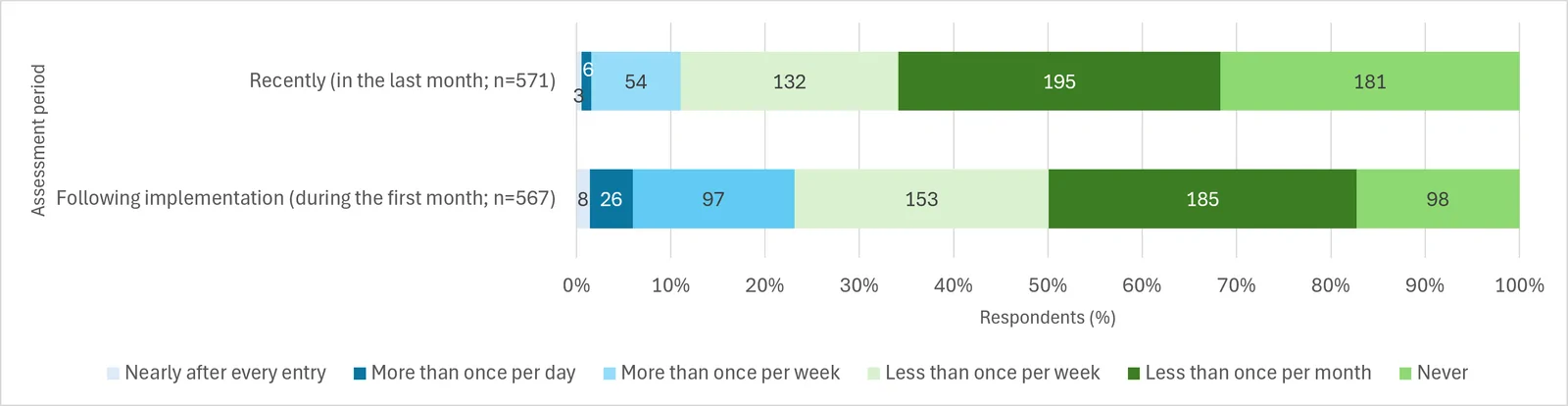
Frequency of investigations and error corrections in the most recent month and following the implementation of the electronic narcotic consumption card reported by nurses and pharmacists.

Nurses and pharmacists reported conducting more investigations and error corrections immediately after eNCC implementation than during the most recent month (*P*<.001; Figure 3). Practical nurses reported conducting these tasks only rarely. For example, 23.1% (131/567) performed these tasks more than once a week after implementation vs 11% (63/571) in the most recent month. Similarly, the proportion of those who never performed investigations and error corrections decreased from 31.7% (181/571) to 17.3% (98/567) from after implementation to the most recent month. Ward pharmacists conducted more investigations and corrections than registered nurses and other pharmacists both after implementation (nurses: *P*<.001; other pharmacists: *P*=.001) and in the most recent month (nurses: *P*<.001; other pharmacists: *P*=.002).

Several statistically significant differences in UX were observed across professional groups (Multimedia Appendix 2). Physicians were less likely than registered nurses (*P*=.02) and ward pharmacists (*P*=.002) to believe that the eNCC prevented drug abuse and diversion. They also rated its impact on improving controlled substance surveillance lower than registered nurses (*P*=.03) and ward pharmacists (*P*=.004). Registered nurses more often agreed that the eNCC facilitated physicians’ verification signatures compared with physicians (*P*=.004) and ward pharmacists (*P*<.001). Practical nurses, compared with registered nurses, considered the eNCC easier to use (*P*=.004); more effective in preventing drug abuse and diversion (*P*=.02); and more effortless to use for liquid oral preparations, infusions, and injections (*P*=.02; Multimedia Appendices 2 and 3). Ward pharmacists were less likely than registered nurses to report that the eNCC reduced investigations and error corrections for liquids (*P*=.049; Multimedia Appendix 3) but more often agreed that it accelerated the documentation process for solids compared with other pharmacists (*P*=.02).

Professional experience significantly influenced UX. HCPs with 0 to 5 years of experience reported learning to use the eNCC more quickly than those with over 15 years of experience (*P*=.003) and were more likely to consider the support and instructions sufficient (*P*=.02). Those with over 15 years of experience perceived less improvement in documentation speed for tablets, capsules, and transdermal patches compared with those with 6 to 15 years of experience (*P*=.005). Experience in the current working unit also mattered: HCPs with 0 to 5 years of experience reported faster learning (*P*=.009) and greater perceived improvement in controlled substance surveillance reliability (*P*=.048) than those with over 15 years of experience. Years of EHR use did not substantially affect UX.

All respondents were asked how likely they would be to recommend the eNCC to HCPs in other hospital wards. Of 563 respondents, 25.8% (n=145) were detractors, 30.6% (n=172) were passively satisfied, and 43.7% (n=246) were promoters, resulting in an NPS of 18. Although UX differed significantly across professional groups, no significant differences in NPS were observed.

A total of 30.7% (184/599) of respondents answered the open-ended question (physicians: 6/184, 3.3%; registered nurses: 158/184, 85.9%; ward pharmacists: 13/184, 7.1%; other pharmacists: 7/184, 3.8%). The most frequently reported themes (n>30) were problematic waste entries, general satisfaction or dissatisfaction with the eNCC, exceptions and errors, and challenging workflows for liquids. Problematic waste entries referred to documentation considered complex, illogical, and hard to remember. Satisfaction related to the eNCC’s adequacy and clear surveillance features, whereas dissatisfaction was linked to slower work pace. Exceptions and errors involved investigations and corrections perceived as complex and time-consuming. Documentation of liquids was also viewed as demanding and time intensive. Detailed results are reported in a separate article and were not the focus of this paper.

## Discussion

### Overall Views on the eNCC Process

This study confirmed that HCPs generally viewed the eNCC process positively, reinforcing earlier findings that it supports reliable real-time documentation of controlled substances and improves efficiency, particularly for solid dosage forms [12]. Among respondents, 80.9% (484/598) opposed returning to paper-based workflows, indicating broad acceptance despite some persistent challenges. However, the NPS was 18, reflecting a polarized user base with both strong promoters and detractors [34]. Because the NPS classifies ratings of 9 and 10 as promoters and ratings of 0 to 6 as detractors and excludes ratings of 7 and 8 from the final scoring, it can be considered a rather strict metric and is therefore best used as a complementary measure rather than a comprehensive indicator on its own [38]. The moderately positive NPS score of 18, despite generally favorable Likert-scale responses, may reflect the stringent scoring methodology of the NPS, where moderately satisfied users (scores of 7‐8) do not contribute to the overall score. Open-ended responses highlighted problematic waste entries, slower workflows, and error handling as key concerns, which can be mitigated through system refinements and robust training, both recognized as critical for successful EHR implementations [39,40].

Perceptions varied by dosage form: processes for liquids were rated as less efficient and more cumbersome than those for solids, and the eNCC was reported to reduce investigations and error corrections more effectively for solids compared to liquids. These findings align with prior observations that injection workflows present particular usability challenges [12]. Encouragingly, the frequency of investigations and error corrections declined markedly from implementation to the survey period, suggesting that system familiarity and process stabilization improve performance, consistent with broader evidence from public sector information system implementations [41]. Pharmacists consistently performed more investigations than nurses, underscoring their central role in controlled substance surveillance and their essential contribution within hospital-based health care teams [42].

### Professional Group Differences in UX

Differences emerged between professional groups. Physicians expressed less confidence in the eNCC’s surveillance benefits compared with nurses and pharmacists. This likely reflects their limited interaction with the system, primarily verifying entries rather than performing documentation and error resolution. Previous research shows that physicians’ EHR satisfaction depends on workflow alignment and information accessibility, whereas poor usability is linked to stress and burnout [21,43]. In our study, physicians’ less positive attitudes may have also stemmed from difficulties locating information and limited awareness of other professionals’ responsibilities within the highly role-specific eNCC process. Interview data from our earlier study revealed that physicians may find reports and verification of entries challenging to use [12]. Indeed, prior to the implementation of the eNCC, their role in the controlled substance oversight process comprised manually signing paper cards. The paper-based process is easier to manage without familiarity with the EHR system workflows. However, each entry in the eNCC is based on medication orders and administration in the EHR system [12]. Improved traceability may therefore be less apparent to physicians whose involvement is limited to the verification phase of eNCC surveillance.

Conversely, nurses and pharmacists, who use the eNCC most frequently, reported greater trust in its surveillance benefits. Their familiarity with barcode scanning–based workflows likely contributed to this confidence [12]. Ward pharmacists were the most common professionals who investigated errors, and pharmacists at the hospital pharmacy approved each eNCC before archiving, making them more aware of issues related to the eNCC. Interestingly, practical nurses reported more positive UX than registered nurses. This may reflect their more limited involvement in workflow details related to the eNCC. Practical nurses reported conducting investigations and error corrections only rarely, which may have reduced their exposure to the challenges associated with eNCC. However, other factors such as differences in use frequency, task complexity, or user expectations may also have contributed to the observed differences. These findings underscore the importance of considering role-specific workflows and access rights in interaction design to support multi-professional work and minimize disruptions during digital transitions [44,45].

### Leveraging the EHR-Integrated eNCC in Controlled Substance Surveillance

Embedding controlled substance surveillance within the EHR offers clear advantages. All administrations are recorded in the medication administration record, enabling reconciliation with dispensing data and improving detection of discrepancies [7]. Structured and standardized documentation further enhances data quality and reuse [46], whereas remote access to integrated records supports workflow efficiency [47]. The eNCC consolidates medication orders and administration of controlled substances in a single location [12], which may explain its generally positive reception among HCPs. However, usability depends on aligning documentation requirements with clinical workflows; excessive clicks, fragmented access, or missing data can undermine efficiency. Future development should prioritize these aspects to maximize safety and user satisfaction. Furthermore, although the eNCC was specifically developed for a particular EHR system and within the framework of Finnish legislation, the experiences gained from its implementation may provide valuable insights for similar initiatives in other health care organizations.

### Strengths and Limitations of the Study and Future Research

Our study has several limitations that warrant consideration. First, the response rate (599/5880, 10%‐599/2520, 24% depending on the denominator) was lower than typical benchmarks for online surveys; this may be partly explained by the fact that the invitations were sent to supervisors to forward the survey links instead of directly sending invitations to all HCP end users. The survey link was not personalized, which in principle allowed a single respondent to submit multiple responses. However, the heterogeneity of the responses and their distribution across professional groups suggest that this was unlikely. The respondents represented all professional groups, with proportions comparable to those of the hospital staff overall. Respondents were drawn from all 14 departments that had implemented the eNCC, supporting the representativeness of the study population. Nevertheless, voluntary surveys may be subject to self-selection bias [48], whereby respondents may have constituted users who were more engaged with the eNCC or more confident in using digital tools than nonrespondents. However, the critical comments expressed in the open-ended responses suggest that participation was not restricted exclusively to highly satisfied users. Second, disentangling eNCC-specific tasks from broader EHR-based medication administration is challenging as several steps are largely similar irrespective of NCC modality [12]. Nonetheless, the survey design, grounded in prior observational work, enabled isolation of key differences by dosage form (solids vs liquids), offering meaningful UX insights. Third, our methodological approach relied on perceived usability and workload, which correlate only modestly with actual EHR-related fatigue [49]. Furthermore, retrospective evaluations may have been influenced by peak and end experiences rather than the cumulative number of implementation-related issues encountered [50,51]. However, these findings build on our earlier observational study [12], and further observations in the expanded deployment context would strengthen triangulation.

Instead of relying on standardized usability questionnaires [16,52-55] or unvalidated commercial instruments [56], we developed a study-specific survey tool. This approach was informed by our previous work [12], which provided an in-depth understanding of the medication workflows associated with the eNCC process. This knowledge allowed us to explicitly account for differences between workflows for solid and liquid dosage forms. Importantly, we recognized that the implementation of the eNCC could have divergent effects, potentially either accelerating or decelerating the documentation of controlled substances. Similarly, the frequency of errors in controlled substance surveillance could plausibly increase or decrease following eNCC implementation. As we used a study-specific questionnaire, direct comparisons with studies using standardized usability measures are not possible. However, the aim was to compare workflows rather than evaluate usability.

Future studies should build on our findings in several ways. Repeating observational work would be valuable as the number of wards using the eNCC has increased substantially since our initial study [12], enabling richer analyses of workflows in diverse contexts. Our survey focused solely on the eNCC process, and without a concurrent paper-based control group, we cannot directly compare UX across modalities. However, a remarkable portion of HCPs (484/598, 80.9%) were unwilling to return to a paper-based process. Therefore, future research should focus on the usability, workload, and error detection of the eNCC while considering role-based access rights and responsibilities. In addition, prospective research on the applicability of the eNCC process in different care settings, such as opioid substitution treatment units, could enhance surveillance practices.

### Conclusions

The eNCC process was generally viewed positively by HCPs. It was preferred over traditional paper-based workflows despite some concerns such as problematic waste entries and challenges in error handling. The electronic process was perceived as easier to manage for solid dosage forms than for liquids. Addressing the usability concerns will be essential for optimizing the process efficiency and robustness as well as user satisfaction in the health care setting. Furthermore, the research highlights the importance of understanding the varying levels of interaction with the eNCC among different professional roles. Ward pharmacists played a central role in conducting more investigations and corrections than nurses responsible for the administration of controlled substances, emphasizing their critical role in controlled substance surveillance within health care teams.

## Supplementary material

10.2196/94815 Multimedia Appendix 1 Survey questionnaire.

10.2196/94815 Multimedia Appendix 2 User experience of surveyed health care professionals regarding the electronic narcotic consumption card process in general.

10.2196/94815 Multimedia Appendix 3 User experience of nurses and pharmacists regarding the electronic narcotic consumption card process for solids (tablets, capsules, and transdermal patches) and liquids (liquid oral preparations, injections, and infusions).

10.2196/94815 Checklist 1 CROSS checklist.

## Abbreviations

CROSS: Consensus-Based Checklist for Reporting of Survey Studies
EHR: electronic health record
eNCC: electronic narcotic consumption card
HCP: health care professional
NCC: narcotic consumption card
NPS: net promoter score
UX: user experience
