# Supplementary material for: User Experiences of Electronic Controlled Substance Surveillance Among Health Care Professionals in a Large Academic Hospital: Cross-Sectional Survey
Source: J Med Internet Res. 2026 Aug 26;28:e94815. doi: 10.2196/94815 (PMC13509440; doi:10.2196/94815)
Supplement: Supplementary file 1 [file jmir-v28-e94815-s001.docx]

*Page 1*

**Kysely sähköisen huumausaineen kulutuskortin käytöstä hoitoyksiköissä**

Kyselyn tavoitteena on selvittää sähköistä huumausaineen kulutuskorttia (sähköinen kulutuskortti) käyttävien terveydenhuollon ammattilaisten näkemyksiä sähköisestä kulutuskortista. Kyselyssä kerättyä aineistoa käytetään kansainvälisen tutkimusjulkaisun laatimisessa. Lisäksi yhteenveto tutkimuksen tuloksista ja kehitysehdotuksista raportoidaan Apotille. Kysely koostuu taustakysymyksistä ja sähköisen kulutuskortin käytettävyyteen ja käyttöön liittyvistä kysymyksistä. Vastaaminen vie noin 5–10 minuuttia riippuen avoimeen vastaukseen käytetystä ajasta.

Kyselyyn vastataan nimettömästi ja vastaaminen on vapaaehtoista. Vastauksia yhdistämällä on kuitenkin mahdollista tunnistaa vastaaja, minkä vuoksi aineistosta muodostuu henkilörekisteri. Tutkimuksen tulokset raportoidaan kaikissa vaiheissa niin, että yksittäistä vastaajaa ei voida tunnistaa. Vastaaminen kyselyyn katsotaan tietoiseksi suostumukseksi. Voit halutessasi pyytää tutkimuksen tietosuojaselosteen tutkimuksen johtavalta tutkijalta: FaT, erikoisproviisori Hanna Tolonen, HUS Apteekki (hanna.tolonen@hus.fi, p. 050 5705371).

HUS Apteekin tutkijat vastaavat kyselyn toteuttamisesta. Tutkimus toteutetaan yhteistyössä Aalto-yliopiston ja Helsingin yliopiston kanssa.

Vastaamalla "Kyllä, haluan vastata kyselyyn sähköisen huumausaineen kulutuskortin käytöstä osastolla." sallin vastausten käyttämisen tutkimustarkoituksiin.

Kyllä, haluan vastata kyselyyn sähköisen huumausaineen kulutuskortin käytöstä osastolla.

Ei, en halua vastata kyselyyn

**TAUSTATIEDOT**

Ammattisi?

Lääkäri

Sairaanhoitaja

Lähihoitaja

Osastofarmaseutti / klinikkaproviisori

Farmaseutti / proviisori HUS Apteekissa

Muu

Mikä on ammattisi?

__________________________________

Missä sairaalassa olet pääsääntöisesti

Haartmanin sairaala

työskennellyt käyttäessäsi sähköistä

Hyvinkään sairaala

kulutuskorttia

Iho- ja allergiasairaala

Jorvin sairaala

Kirurginen sairaala

Lohjan sairaala

Malmin sairaala

Meilahden kolmiosairaala

Meilahden siltasairaala

Meilahden tornisairaala

Peijaksen sairaala

Porvoon sairaala

Psykiatriakeskus

Puistosairaala

Raaseporin sairaala

Silmä-korvasairaala

Syöpäkeskus

Uusi lastensairaala

Muu (esimerkiksi Diagnostiikkakeskus, HUS Apteekki)

Missä tulosyksikössä olet pääsääntöisesti

Akuutti

työskennellyt käyttäessäsi sähköistä

Lasten ja nuorten sairaudet

kulutuskorttia?

Leikkaussalit ja tehohoito

Lähisairaaloiden medisiiniset palvelut

Lähisairaaloiden operatiiviset palvelut

Naistentaudit ja synnytykset

Neurokeskus

Psykiatria

Pää- ja kaulakeskus

Sisätaudit ja kuntoutus

Sydän- ja keuhkokeskus

Syöpäkeskus

Tukielin- ja plastiikkakirurgia

Tulehduskeskus

Vatsakeskus

HUS Apteekki

Kuinka kauan olet työskennellyt nykyisessä

vuotta

0–5

ammatissasi?

6–10

vuotta

11–15

vuotta

16–20

vuotta

21

vuotta tai enemmän

Kuinka kauan olet työskennellyt nykyisessä

0–5

vuotta

työyksikössäsi?

6–10

vuotta

11–15

vuotta

16–20

vuotta

21

vuotta tai enemmän

Kuinka kauan olet käyttänyt Apotti-järjestelmää?

Alle 6 kuukautta

6–12

kuukautta

1–3

vuotta

Yli 3 vuotta (Apotin käyttöönotot vuosina

2018–2020)

**TARKENTAVAT KYSYMYKSET SÄHKÖISESTÄ HUUMAUSAINEEN KULUTUSKORTISTA**

**Arvioi seuraavia väittämiä**

| Täysin eri mieltä | Jokseenkin eri mieltä | En samaa enkä eri mieltä | Jokseenkin samaa mieltä | Täysin samaa mieltä |
| --- | --- | --- | --- | --- |

Käyttökokemukseni perusteella haluan palata käyttämään paperista huumausaineen kulutuskorttia.

Sähköisen kulutuskortin käyttöönottovaiheessa tuki ja apu oli riittävää.

Opin nopeasti käyttämään sähköistä kulutuskorttia.

Tällä hetkellä sähköisen kulutuskortin käytön tuki ja ohjeistus on riittävää.

Koen, että sähköisen kulutuskortin käyttö parantaa huumausainelääkkeiden käytön seurantaa.

Koen, että sähköinen kulutuskortin käyttö ehkäisee huumausainelääkkeiden väärinkäyttöä.

**Arvioi seuraavia väittämiä. Sähköisen kulutuskortin käyttö...**

| Täysin eri mieltä | Jokseenkin eri mieltä | En samaa enkä eri mieltä | Jokseenkin samaa mieltä | Täysin samaa mieltä |
| --- | --- | --- | --- | --- |

... on sujuvaa työyksikössäni.

... helpottaa lääkärin tekemää huumausainekorttien allekirjoittamista.

... ehkäisee huumausainelääkkeiden väärinkäyttötilanteita.

... parantaa huumausainelääkkeiden käytön seurannan luotettavuutta.

**Arvioi tilannetta KIINTEIDEN huumausainelääkkeiden (tabletit, kapselit, laastarit) osalta:**

| Täysin eri mieltä | Jokseenkin eri mieltä | En samaa enkä eri mieltä | Jokseenkin samaa mieltä | Täysin samaa mieltä |
| --- | --- | --- | --- | --- |

Sähköisen kulutuskortin käyttö on sujuvaa omassa työssäni.

Sähköinen kulutuskortti on nopeuttanut huumausainekirjausten tekemistä.

Sähköinen kulutuskortti on vähentänyt jälkikäteen tehtävää kirjausten selvittely- ja korjaustyötä.

Koen, että yksikössäni sähköisen kulutuskortin käyttö sujuu hyvin.

Sähköisen kulutuskortin käyttö on hitaampaa kuin paperisen kulutuskortin käyttö.

**Arvioi tilannetta NESTEMÄISTEN eli käyttökuntoon saatettavien huumausainelääkkeiden (oraaliliuokset, infuusiot, injektiot) osalta:**

| Täysin eri mieltä | Jokseenkin eri mieltä | En samaa enkä eri mieltä | Jokseenkin samaa mieltä | Täysin samaa mieltä |
| --- | --- | --- | --- | --- |

Sähköisen kulutuskortin käyttö on sujuvaa omassa työssäni.

Sähköinen kulutuskortti on nopeuttanut huumausainekirjausten tekemistä.

Sähköinen kulutuskortti on vähentänyt jälkikäteen tehtävää

kirjausten selvittely- ja korjaustyötä.

Koen, että yksikössäni sähköisen kulutuskortin käyttö sujuu hyvin.

Sähköisen kulutuskortin käyttö on hitaampaa kuin paperisen kulutuskortin käyttö.

**Kuinka usein olet selvitellyt tai korjannut huumausainekirjauksia JÄLKIKÄTEEN sähköisestä kulutuskortista (esim. kirjaus ei näy oikein kulutuskortilla)?**

| Lähes jokaisen  kirjauksen jälkeen | Useammin  kuin kerran päivässä | Useammin  kuin kerran  viikossa | Harvemmin kuin kerran  viikossa | Harvemmin kuin kerran kuukaudessa | En lainkaan |
| --- | --- | --- | --- | --- | --- |

Käyttöönoton jälkeen (ensimmäisen kuukauden aikana)

Viime aikoina (viimeisen kuukauden aikana)

**SUOSITTELU JA PALAUTE**

Miten todennäköisesti (asteikko 0–10) suosittelisit sähköisen huumausaineen kulutuskortin käyttöä muissa hoitoyksiköissä työskenteleville kollegoillesi?

___________________________________________________________________________________________________________

Voit halutessasi antaa palautetta sähköiseen

kulutuskorttiin tai sen kehittämiseen liittyen.

__________________________________________
