# Supplementary material for: User Experiences of Electronic Controlled Substance Surveillance Among Health Care Professionals in a Large Academic Hospital: Cross-Sectional Survey
Source: J Med Internet Res. 2026 Aug 26;28:e94815. doi: 10.2196/94815 (PMC13509440; doi:10.2196/94815)
Supplement: Supplementary file 2 [file jmir-v28-e94815-s002.docx]

**Table 2.** User experience of surveyed health care professionals regarding the electronic narcotic consumption card (eNCC) process in general.

| **Statements** | **Total respondents, n** | **P value** | **Physicians vs RNs^a^** | | | **Physicians vs WPs^b^** | | | **RNs vs WPs** | | | **RNs vs PNs^c^** | | | **WPs vs OPs^d^** | | |
| --- | --- | --- | --- | --- | --- | --- | --- | --- | --- | --- | --- | --- | --- | --- | --- | --- | --- |
|  |  |  | Physicians, n/N (%) | RNs, n/N (%) | P value | Physicians, n/N (%) | WPs, n/N (%) | P value | RNs, n/N (%) | WPs, n/N (%) | P value | RNs, n/N (%) | PNs, n/N (%) | P value | WPs, n/N (%) | OPs, n/N (%) | P value |
| Based on my user experience on eNCC, I would like to return to use paper-based NCC. | | .28 |  |  | —^e^ |  |  | — |  |  | — |  |  | — |  |  | — |
| Agree | 79/598 (13) |  | 2/21 (10) | 73/510 (14) |  | 2/21 (10) | 1/33 (3) |  | 73/510 (14) | 1/33 (3) |  | 73/510 (14) | 1/11 (9) |  | 1/33(3) | 2/23(9) |  |
| Neutral | 35/598 (6) |  | 1/21 (5) | 32/510 (6) |  | 1/21 (5) | 0/33 (0) |  | 32/510 (6) | 0/33 (0) |  | 32/510 (6) | 1/11 (9) |  | 0/33(0) | 1/23(4) |  |
| Disagree | 484/598 (81) |  | 18/21 (86) | 405/510 (79) |  | 18/21 (86) | 32/33 (97) |  | 405/510 (79) | 32/33 (97) |  | 405/510 (79) | 9/11 (82) |  | 32/33(97) | 20/23(87) |  |
| Technical support was adequate during the implementation of eNCC. | | .13 |  |  | — |  |  | — |  |  | — |  |  | — |  |  | — |
| Agree | 340/595 (57) |  | 14/21 (67) | 282/509 (55) |  | 14/21 (67) | 21/33 (64) |  | 282/509 (55) | 21/33 (64) |  | 282/509 (55) | 7/11 (64) |  | 21/33(64) | 16/21(76) |  |
| Neutral | 87/595 (15) |  | 3/21 (14) | 73/509 (14) |  | 3/21 (14) | 6/33 (18) |  | 73/509 (14) | 6/33 (18) |  | 73/509 (14) | 3/11 (27) |  | 6/33(18) | 2/21(10) |  |
| Disagree | 168/595 (28) |  | 4/21 (19) | 154/509 (30) |  | 4/21 (19) | 6/33 (18) |  | 154/509 (30) | 6/33 (18) |  | 154/509 (30) | 1/11 (9) |  | 6/33(18) | 3/21(15) |  |
| I learned to use eNCC easily. | | .26 |  |  | — |  |  | — |  |  | — |  |  | — |  |  | — |
| Agree | 449 |  | 15/21 (71) | 381/510 (75) |  | 15/21 (71) | 24/33 (73) |  | 381/510 (75) | 24/33 (73) |  | 381/510 (75) | 10/11 (91) |  | 24/33(73) | 19/23(83) |  |
| Neutral | 47 |  | 2/21 (10) | 41/510 (8) |  | 2/21 (10) | 3/33 (9) |  | 41/510 (8) | 3/33 (9) |  | 41/510 (8) | 1/11 (9) |  | 3/33(9) | 0/23(0) |  |
| Disagree | 102 |  | 4/21 (19) | 88/510 (17) |  | 4/21 (19) | 6/33 (18) |  | 88/510 (17) | 6/33 (18) |  | 88/510 (17) | 0/11 (0) |  | 6/33(18) | 4/23(17) |  |
| Support and instructions for using eNCC are currently sufficient. | | .03^f^ |  |  | >.99 |  |  | .39 |  |  | .41 |  |  | .41 |  |  | .34 |
| Agree | 434/597 (73) |  | 14/21 (67) | 363/509 (71) |  | 14/21 (67) | 30/33 (91) |  | 363/509 (71) | 30/33 (91) |  | 363/509 (71) | 11/11 (100) |  | 30/33(91) | 16/23(70) |  |
| Neutral | 66/597 (11) |  | 4/21 (19) | 59/509 (12) |  | 4/21 (19) | 2/33 (6) |  | 59/509 (12) | 2/33 (6) |  | 59/509 (12) | 0/11 (0) |  | 2/33(6) | 1/23(4) |  |
| Disagree | 97/597 (16) |  | 3/21 (14) | 87/509 (17) |  | 3/21 (14) | 1/33 (3) |  | 87/509 (17) | 1/33 (3) |  | 87/509 (17) | 0/11 (0) |  | 1/33(3) | 6/23(26) |  |
| In my opinion, eNCC improves the surveillance of controlled substances. | | .004 |  |  | .01 |  |  | .002 |  |  | .53 |  |  | >.99 |  |  | .70 |
| Agree | 432/597 (72) |  | 10/21 (48) | 368/509 (72) |  | 10/21 (48) | 31/33 (94) |  | 368/509 (72) | 31/33 (94) |  | 368/509 (72) | 8/11 (73) |  | 31/33(94) | 15/23(65) |  |
| Neutral | 96/597 (16) |  | 4/21 (19) | 83/509 (16) |  | 4/21 (19) | 1/33 (3) |  | 83/509 (16) | 1/33 (3) |  | 83/509 (16) | 3/11 (27) |  | 1/33(3) | 5/23(22) |  |
| Disagree | 69/597 (12) |  | 7/21 (33) | 58/509 (11) |  | 7/21 (33) | 1/33 (3) |  | 58/509 (11) | 1/33 (3) |  | 58/509 (11) | 0/11 (0) |  | 1/33(3) | 3/23(13) |  |
| In my opinion, eNCC prevents drug abuse and diversion. | | <.001 |  |  | .02 |  |  | .002 |  |  | .61 |  |  | .37 |  |  | .53 |
| Agree | 373/599 (62) |  | 7/21 (33) | 314/511 (61) |  | 7/21 (33) | 29/33 (88) |  | 314/511 (61) | 29/33 (88) |  | 314/511 (61) | 9/11 (82) |  | 29/33(88) | 14/23(61) |  |
| Neutral | 139/599 (23) |  | 7/21 (33) | 122/511 (24) |  | 7/21 (33) | 3/33 (9) |  | 122/511 (24) | 3/33 (9) |  | 122/511 (24) | 2/11 (18) |  | 3/33 (9) | 5/23(22) |  |
| Disagree | 87/599 (15) |  | 7/21 (33) | 75/511 (15) |  | 7/21 (33) | 1/33 (3) |  | 75/511 (15) | 1/33 (3) |  | 75/511 (15) | 0/11 (0) |  | 1/33 (3) | 4/23(17) |  |
| The use of eNCC is effortless at my ward. | | .002 |  |  | >.99 |  |  | >.99 |  |  | >.99 |  |  | .004 |  |  | >.99 |
| Agree | 442/597 (74) |  | 14/20 (70) | 379/511 (74) |  | 14/20 (70) | 24/33 (73) |  | 379/511 (74) | 24/33 (73) |  | 379/511 (74) | 11/11 (100) |  | 24/33 (73) | 14/22(64) |  |
| Neutral | 52/597 (9) |  | 5/20 (25) | 45/511 (9) |  | 5/20 (25) | 2/33 (6) |  | 45/511 (9) | 2/33 (6) |  | 45/511 (9) | 0/11 (0) |  | 2/33 (6) | 0/22(0) |  |
| Disagree | 103/597 (17) |  | 1/20 (5) | 87/511 (17) |  | 1/20 (5) | 7/33 (21) |  | 87/511 (17) | 7/33 (21) |  | 87/511 (17) | 0/11 (0) |  | 7/33 (21) | 8/22(36) |  |
| The use of eNCC facilitates physicians’ verification signature. | | <.001 |  |  | .004 |  |  | >.99 |  |  | <.001 |  |  | >.99 |  |  | >.99 |
| Agree | 222/588 (38) |  | 16/21 (76) | 162/502 (32) |  | 16/21 (76) | 26/33 (79) |  | 162/502 (32) | 26/33 (79) |  | 162/502 (32) | 5/11 (45) |  | 26/33 (79) | 13/21(62 |  |
| Neutral | 352/588 (60) |  | 4/21 (19) | 332/502 (66) |  | 4/21 (19) | 4/33 (12) |  | 332/502 (66) | 4/33 (12) |  | 332/502 (66) | 6/11 (55) |  | 4/33 (12) | 6/21(29) |  |
| Disagree | 14/588 (2) |  | 1/21 (5) | 8/502 (2) |  | 1/21 (5) | 3/33 (9) |  | 8/502 (2) | 3/33 (9) |  | 8/502 (2) | 0/11 (0) |  | 3/33 (9) | 2/21(10) |  |
| The use of eNCC prevents possible situations for drug abuse and diversion. | | .001 |  |  | .07 |  |  | .048 |  |  | >.99 |  |  | .02 |  |  | >.99 |
| Agree | 356/593 (60) |  | 7/20 (35) | 301/508 (59) |  | 7/20 (35) | 25/33 (76) |  | 301/508 (59) | 25/33 (76) |  | 301/508 (59) | 10/11 (91) |  | 25/33 (76) | 13/21(62) |  |
| Neutral | 169/593 (28) |  | 8/20 (40) | 147/508 (29) |  | 8/20 (40) | 7/33 (21) |  | 147/508 (29) | 7/33 (21) |  | 147/508 (29) | 1/11 (9) |  | 7/33 (21) | 6/21(29 |  |
| Disagree | 68/593 (11) |  | 5/20 (25) | 60/508 (12) |  | 5/20 (25) | 1/33 (3) |  | 60/508 (12) | 1/33 (3) |  | 60/508 (12) | 0/11 (0) |  | 1/33 (3) | 2/21(10) |  |
| The use of eNCC improves the reliability of controlled substances surveillance. | | <.001 |  |  | .03 |  |  | .004 |  |  | .73 |  |  | .23 |  |  | .20 |
| Agree | 439/594 (74) |  | 12/21 (57) | 371/507 (73) |  | 12/21 (57) | 30/33 (91) |  | 371/507 (73) | 30/33 (91) |  | 371/507 (73) | 10/11 (91) |  | 30/33 (91) | 16/22(73) |  |
| Neutral | 105/594 (18) |  | 5/21 (24) | 94/507 (19) |  | 5/21 (24) | 3/33 (9) |  | 94/507 (19) | 3/33 (9) |  | 94/507 (19) | 1/11 (9) |  | 3/33 (9) | 2/22(9) |  |
| Disagree | 50/594 (8) |  | 4/21 (19) | 42/507(8) |  | 4/21(19) | 0/33(0) |  | 42/507(8) | 0/33 (0) |  | 42/507 (8) | 0/11 (0) |  | 0/33 (0) | 4/22(18) |  |

^a^ Registered nurses

^b^ Ward pharmacists

^c^ Practical nurses

^d^ Other pharmacists

^e^ No pairwise comparisons were conducted because the overall result was not statistically significant.

^f^ Statistically significant difference between groups
