# Supplementary material for: User Experiences of Electronic Controlled Substance Surveillance Among Health Care Professionals in a Large Academic Hospital: Cross-Sectional Survey
Source: J Med Internet Res. 2026 Aug 26;28:e94815. doi: 10.2196/94815 (PMC13509440; doi:10.2196/94815)
Supplement: Supplementary file 3 [file jmir-v28-e94815-s003.docx]

**Table 3.** User experience of nurses and pharmacists regarding the electronic narcotic consumption card (eNCC) process for solids (tablets, capsules, and transdermal patches) and liquids (liquid oral preparations, injections, and infusions).

| **Statements** | **Total respondents, n** | **P value** | **RNs^a^ vs WPs^b^** | | | **RNs vs PNs^c^** | | | **WPs vs OPs^d^** | | |
| --- | --- | --- | --- | --- | --- | --- | --- | --- | --- | --- | --- |
|  |  |  | RNs, n/N (%) | WPs, n/N (%) | P value | RNs, n/N (%) | PNs, n/N (%) | P value | WPs, n/N (%) | OPs, n/N (%) | P value |
| Solids: eNCC use is effortless in my daily work. | | .25 |  |  | —^e^ |  |  | — |  |  | — |
| Agree | 491/578 (85) |  | 431/511 (84) | 30/33 (91) |  | 431/511 (84) | 11/11 (100) |  | 30/33 (91) | 19/23 (83) |  |
| Neutral | 32/578 (6) |  | 30/511 (6) | 2/33 (6) |  | 30/511 (6) | 0/11 (0) |  | 2/33 (6) | 0/23 (0) |  |
| Disagree | 55/578 (10) |  | 50/511 (10) | 1/33 (3) |  | 50/511 (10) | 0/11 (0) |  | 1/33 (3) | 4/23 (17) |  |
| Liquids: eNCC use is effortless in my daily work. | | .08 |  |  | — |  |  | — |  |  | — |
| Agree | 418/578 (72) |  | 371/511 (73) | 23/33 (70) |  | 371/511 (73) | 10/11 (91) |  | 23/33 (70) | 14/23 (61) |  |
| Neutral | 50/578 (9) |  | 43/511 (8) | 3/33 (9) |  | 43/511 (8) | 1/11 (9) |  | 3/33 (9) | 3/23 (13) |  |
| Disagree | 110/578 (19) |  | 97/511 (19) | 7/33 (21) |  | 97/511 (19) | 0/11 (0) |  | 7/33 (21) | 6/23 (26) |  |
| Solids: eNCC has accelerated the documentation process. | | .02^f^ |  |  | .29 |  |  | >.99 |  |  | .02 |
| Agree | 408/572 (71) |  | 365/510 (72) | 28/33 (85) |  | 365/510 (72) | 7/11 (64) |  | 28/33 (85) | 8/18 (44) |  |
| Neutral | 50/572 (9) |  | 38/510 (7) | 3/33 (9) |  | 38/510 (7) | 2/11 (18) |  | 3/33 (9) | 7/18 (39) |  |
| Disagree | 114/572 (20) |  | 107/510 (21) | 2/33 (6) |  | 107/510 (21) | 2/11 (18) |  | 2/33 (6) | 3/18 (17) |  |
| Liquids: eNCC has accelerated the documentation process. | | .23 |  |  | — |  |  | — |  |  | — |
| Agree | 328/572 (57) |  | 291/510 (57) | 19/33 (58) |  | 291/510 (57) | 9/11 (82) |  | 19/33 (58) | 9/18 (50) |  |
| Neutral | 62/572 (11) |  | 47/510 (9) | 7/33 (21) |  | 47/510 (9) | 1/11 (9) |  | 7/33 (21) | 7/18 (39) |  |
| Disagree | 182/572 (32) |  | 172/510 (34) | 7/33 (21) |  | 172/510 (34) | 1/11 (9) |  | 7/33 (21) | 2/18 (11) |  |
| Solids: eNCC has reduced investigations and error corrections. | | .11 |  |  | — |  |  | — |  |  | — |
| Agree | 250/574 (44) |  | 228/511 (45) | 10/33 (30) |  | 228/511 (45) | 6/11 (55) |  | 10/33 (30) | 6/19 (32) |  |
| Neutral | 133/574 (23) |  | 115/511 (23) | 10/33 (30) |  | 115/511 (23) | 4/11 (36) |  | 10/33 (30) | 4/19 (21) |  |
| Disagree | 191/574 (33) |  | 168/511 (33) | 13/33 (39) |  | 168/511 (33) | 1/11 (9) |  | 13/33 (39) | 9/19 (47) |  |
| Liquids: eNCC has reduced investigations and error corrections. | | .001 |  |  | .049 |  |  | .09 |  |  | .51 |
| Agree | 206/574 (36) |  | 184/511 (36) | 7/33 (21) |  | 184/511 (36) | 7/11 (64) |  | 7/33 (21) | 8/19 (42) |  |
| Neutral | 130/574 (23) |  | 122/511 (24) | 3/33 (9) |  | 122/511 (24) | 2/11 (18) |  | 3/33 (9) | 3/19 (16) |  |
| Disagree | 238/574 (41) |  | 205/511 (40) | 23/33 (70) |  | 205/511 (40) | 2/11 (18) |  | 23/33 (70) | 8/19 (42) |  |
| Solids: eNCC use is effortless at my ward. | | .09 |  |  | — |  |  | — |  |  | — |
| Agree | 431/574 (75) |  | 383/508 (75) | 27/33 (82) |  | 383/508 (75) | 9/11 (82) |  | 27/33 (82) | 12/22 (55) |  |
| Neutral | 64/574 (11) |  | 59/508 (12) | 2/33 (6) |  | 59/508 (12) | 2/11 (18) |  | 2/33 (6) | 1/22 (5) |  |
| Disagree | 79/574 (14) |  | 66/508 (13) | 4/33 (12) |  | 66/508 (13) | 0/11 (0) |  | 4/33 (12) | 9/22 (41) |  |
| Liquids: eNCC use is effortless at my ward. | | .003 |  |  | .72 |  |  | .02 |  |  | >.99 |
| Agree | 378/573 (66) |  | 336/508 (66) | 19/33 (58) |  | 336/508 (66) | 10/10 (100) |  | 19/33 (58) | 13/22 (59) |  |
| Neutral | 84/573 (15) |  | 77/508 (15) | 4/33 (12) |  | 77/508 (15) | 0/10 (0) |  | 4/33 (12) | 3/22 (14) |  |
| Disagree | 111/573 (19) |  | 95/508 (19) | 10/33 (30) |  | 95/508 (19) | 0/10 (0) |  | 10/33 (30) | 6/22 (27) |  |
| Solids: eNCC process is less efficient than paper-based NCC process. | | .08 |  |  | — |  |  | — |  |  | — |
| Agree | 146/575 (25) |  | 131/509 (26) | 4/33 (12) |  | 131/509 (26) | 3/11 (27) |  | 4/33 (12) | 8/22 (36) |  |
| Neutral | 72/575 (13) |  | 67/509 (13) | 3/33 (9) |  | 67/509 (13) | 1/11 (9) |  | 3/33 (9) | 1/22 (5) |  |
| Disagree | 357/575 (62) |  | 311/509 (61) | 26/33 (79) |  | 311/509 (61) | 7/11 (64) |  | 26/33 (79) | 13/22 (59) |  |
| Liquids: eNCC process is less efficient than paper-based NCC process. | | .93 |  |  | — |  |  | — |  |  | — |
| Agree | 205/576 (36) |  | 185/510 (36) | 10/33 (30) |  | 185/510 (36) | 4/11 (36) |  | 10/33 (30) | 6/22 (27) |  |
| Neutral | 95/576(16) |  | 82/510(16) | 7/33(21) |  | 82/510(16) | 2/11 (18) |  | 7/33 (21) | 4/22 (18) |  |
| Disagree | 276/576(48) |  | 243/510(48) | 16/33(48) |  | 243/510(48) | 5/11 (45) |  | 16/33 (48) | 12/22 (55) |  |

^a^ Registered nurses

^b^ Ward pharmacists

^c^ Practical nurses

^d^ Other pharmacists

^e^ No pairwise comparisons were conducted because the overall result was not statistically significant.

^f^ Statistically significant difference between groups
